# Short-Term Phantom Recollection in 8–10-Year-Olds and Young Adults

**DOI:** 10.3390/jintelligence11040067

**Published:** 2023-03-30

**Authors:** Marlène Abadie, Manon Rousselle

**Affiliations:** Laboratoire de Psychologie Cognitive, Aix Marseille Université, CNRS, 13331 Marseille, France

**Keywords:** false memory, phantom recollection, working memory, conjoint recognition

## Abstract

Illusory conscious experience of the “presentation” of unstudied material, called phantom recollection, occurs at high levels in long-term episodic memory tests and underlies some forms of false memory. We report an experiment examining, for the first time, the presence of phantom recollection in a short-term working memory (WM) task in 8- to 10-year-old children and young adults. Participants studied lists of eight semantically related words and had to recognize them among unpresented distractors semantically related and unrelated to the studied words after a retention interval of a few seconds. Regardless of whether the retention interval was filled with a concurrent task that interfered with WM maintenance, the false recognition rate for related distractors was very high in both age groups, although it was higher in young adults (47%) than children (42%) and rivaled the rate of target acceptance. The conjoint recognition model of fuzzy-trace theory was used to examine memory representations underlying recognition responses. In young adults, phantom recollection underpinned half of the false memories. By contrast, in children, phantom recollection accounted for only 16% of them. These findings suggest that an increase in phantom recollection use may underlie the developmental increase in short-term false memory.

## 1. Introduction

One of the most intriguing findings of the past two decades of research in human memory is that people can report consciously reexperiencing events that never actually happened. The term phantom recollection has been proposed to cover these subjectively compelling false memories (Brainerd et al. 2001, 2003). A high level of phantom recollection has been reported in the Deese–Roediger–McDermott paradigm (DRM, Deese 1959; Roediger and McDermott 1995). Participants study long lists of words (e.g., 12 to 15 words) that share a common meaning (e.g., “note, sound, piano, sing, radio, band,” etc.) and are all associated with an unpresented critical word (e.g., “music”). When the critical word is presented in a subsequent recognition test, a high false alarm rate is observed, often approaching the acceptance rate of studied words (e.g., 81% in Roediger and McDermott 1995, Exp. 2; see also, Brainerd et al. 2001; Payne et al. 1996). Moreover, false recognition of critical distractors is accompanied by high levels of phantom recollective experience. For example, studies have shown that false memories are associated with as many *remember* judgments as true memories (e.g., 58% in Roediger and McDermott 1995, Exp. 2), which reflects the fact that participants have the illusion of being able to retrieve the particular experiences that accompanied the “presentation” of the critical distractor (see also, Gallo et al. 2001; Payne et al. 1996). In DRM studies, long lists of related words have to be remembered, and the memory test usually takes place minutes, hours, or even days after the study phase. However, recent studies have revealed that false memories can also occur in working memory (WM) tasks for lists of only a few items when a short 4-s interval was given between study and test (Abadie and Camos 2019; Atkins and Reuter-Lorenz 2008). This finding was replicated with children as young as 4 years old (Rousselle et al. 2022). The aim of the present study is to examine whether these short-term false memories observed in young children and adults can be accompanied by such vivid recollection as long-term false memories in the DRM paradigm. 

Fuzzy-trace theory (FTT, Brainerd and Reyna 2018), an opponent process theory, provides one of the most prominent accounts of false memory. It assumes that people store two dissociated representations of experience, verbatim and gist traces, which sometimes reinforce each other and sometimes oppose each other. FTT proposes that when participants encode a list containing semantic associates such as “note, sound, piano, sing,” and so on, they store verbatim traces of the surface forms of individual words’ presentation and associated contextual details, as well as gist traces of their semantic content, particularly the meaning relations that connect them (e.g., “music” words). In a subsequent memory test, verbatim retrieval supports true memory for list words and suppresses false memory for distractors, whereas gist retrieval supports both true and false memory. Verbatim retrieval is assumed to provoke feelings of explicit recollection. Although gist retrieval typically provokes feelings of familiarity (“I know I’ve heard words related to music, but I can’t remember which ones”), it may also generate phantom recollection of specific details of word presentation (“I remember hearing the word music, it was the second word on the study list”) under certain conditions (e.g., Brainerd et al. 1999, 2001). Phantom recollection typically occurred when (a) many items that share meaning are studied, like in the DRM paradigm, so that gist memories of those meanings are very strong, and (b) distractors that are excellent retrieval cues for those gist memories, such as critical distractors in the DRM paradigm, are administered at test (Brainerd and Reyna 1998; Brainerd et al. 2001). Accordingly, false memories produced in such a paradigm have been associated with high confidence (Kim and Cabeza 2007; Roediger and McDermott 1995), remember judgments (Gallo et al. 2001; Israel and Schacter 1997; Pesta et al. 2001; Roediger and McDermott 1995), and retrieval of item-specific details (Geraci and McCabe 2006; Payne et al. 1996). 

Brainerd et al. (1999, 2001) developed the conjoint recognition (CR) procedure, whereby remembering phenomenologies can be extracted directly from memory responses rather than indirectly from introspective reports such as confidence or remember-know judgments. The procedure is that of a standard memory-recognition experiment, except that participants respond to recognition tests under three sets of simple instructions: verbatim instructions, in which participants are told to accept only studied items and reject all unpresented distractors; gist instructions, in which they are told to accept only unpresented distractors that share meaning with studied items and reject all other probes; and verbatim plus gist instructions, in which they are told to accept both targets and semantically related distractors but reject unrelated distractors. Differences in recognition performance between these instructions are then used to measure the contribution of verbatim memory, gist memory (vague gist-based similarity), phantom recollection, and guessing. Applying the CR methodology to the DRM paradigm, Brainerd et al. (2001) showed that false recognition of critical distractors in standard recognition tests (verbatim condition) was overwhelmingly due to phantom recollection rather than vague gist memory (75% vs. 24%, respectively, in Exp. 2), and phantom recollection continued to predominate over gist memory as a basis for critical distractor false alarms after 1 week. A simplification of the CR procedure was then introduced by Stahl and Klauer (2009). Instead of asking participants to respond to recognition probes according to three instructions, they were asked to classify each probe as either studied, related, or unrelated. The simplified CR procedure provides valid estimates of the different memory and guessing parameters, and it is more efficient than the original paradigm because it requires only one group of participants instead of three. Using this procedure, Stahl and Klauer (2009) obtained phantom recollection for DRM-like lists from which eight related words were presented during the study phase but not for lists from which a single word was presented. This finding provides further evidence that phantom recollection occurs when several strongly related items are studied. Finally, reliable phantom recollection estimates have also been obtained for false recall of related items using DRM lists (Brainerd et al. 2003; Marche and Brainerd 2012).

Another advantage of the CR procedure is that it can be used to obtain reliable measures of remembering phenomenologies underlying recognition performance in young children. This procedure does not require introspective reports such as the remember-know procedure, which is more appropriate for young children who may have difficulty understanding and reporting their own mental states. The large archive of DRM research demonstrates that as development unfolds, critical distractors (and other semantically related distractors) are increasingly misremembered as being studied items (e.g., Brainerd and Reyna 2012, 2015; Chang and Brainerd 2021, for reviews). According to the FTT, improvements in gist memory with age increase the tendency to accept related distractors as being studied, and parallel improvements in verbatim memory are ineffective in suppressing this tendency (Brainerd et al. 2011, 2018). However, to date, only a few studies have examined the developmental trend of phantom recollection using the CR procedure. Brainerd et al. (2004, Exp. 1), using DRM lists, showed an increase in false alarm rates for critical distractors between 7- and 11-year-olds, accompanied by an increase in phantom recollection (i.e., 13% to 31%, respectively). Phantom recollection (and false alarm rates) remained stable between 11 and 14 years. Vague-gist-based similarity retrieval for critical distractors was not affected by age. Phantom recollection did not increase with age for other types of semantically related distractors that, unlike critical distractors, do not produce high levels of false memory responses. Odegard et al. (2008), using DRM lists for which each word was paired with an associate that made its meaning either congruent or incongruent with the critical distractor, found no reliable increase in phantom recollection in response to critical distractors between 11-year-olds and young adults (*M*_age_ = 23.7 years) in either condition (i.e., 5% to 19% for children and adults, respectively, in the congruent condition). No age-related increase in vague gist retrieval was observed in response to these distractors. In sum, in young adults, phantom recollection appears to underlie false memories of distractors that have been repeatedly cued by studied items. Although research on its development is scarce, the few studies that have examined the phenomenon have shown an increase from age 7 to early adolescence in response to critical distractors in the DRM paradigm. 

Turning to WM, many studies have shown that false memories also occurred in short-term WM tasks. Atkins and Reuter-Lorenz (2008) were the first to report a fairly high level of critical distractor false alarms (31%) following the study of 4-word DRM lists and a 3- to 4-s retention interval. Flegal et al. (2010, Exp. 2; Flegal and Reuter-Lorenz 2014, Exp.2) used the same paradigm and examined whether false memory responses in short-term tests are accompanied by illusory recollection as they are in the classic DRM paradigm. Participants were asked to indicate for each recognition probe whether they recollected something distinctive about its study (the *remember* response), whether they recognized it without retrieving specific details of its study (the *know* response), or whether their response was a guess. The results of both experiments revealed that among false alarms to related distractors (17.5% and 16% on average, in each experiment, respectively), a significant percentage was attributed to the *remember* response (36% and 25% on average, respectively). Although smaller than that reported in classic DRM studies, it did not differ significantly between the short-term test and a long-term test performed at the end of the experiments (27% and 32% on average, in each experiment, respectively). These findings suggest that short-term false memories as well as long-term false memories might be accompanied by vivid yet illusory recollection of the presentation of related distractors. Abadie and Camos (2019) conducted a series of experiments using the simplified CR procedure in a WM task to dissociate the contributions of verbatim memory, gist memory, and guessing processes to recognition performance. The highest rate of false recognition of related distractors was obtained when maintenance of information in WM through articulatory rehearsal, a mechanism that operates by phonological repetition of items to be remembered (Camos 2015, 2017), was prevented (31% on average) compared to when it was not (11.5% on average). This increase in false memories when articulatory rehearsal was prevented was accompanied by a drastic decrease in verbatim memory retrieval, that is, the tendency for a related item to provoke mental reinstatement of the presentation of the corresponding studied items (e.g., when the distractor “rhythm” provokes recollection of targets such as “note” or “sound”). Verbatim retrieval probability was zero, whereas it was equal to 39% on average when articulatory rehearsal was available. Therefore, when articulatory rehearsal was prevented, responses were mainly based on gist retrieval, which was fairly high (78% on average), leading participants to make more false memories. Unfortunately, in these experiments, estimates of gist retrieval were not distinguished in terms of vague gist memory and phantom recollection, and therefore the contribution of phantom recollection to short-term false memories remains unknown.

The present study aimed to examine whether phantom recollection underlies short-term false memories in 8–10-year-olds and young adults. Only one study has investigated the occurrence of false memories in a WM task in 4- and 8-year-olds using the simplified CR procedure (Rousselle et al. 2022). False memories occurred in both age groups but were few (about 10%). They were primarily underpinned by gist memory retrieval in the absence of strong verbatim traces that could counteract gist-based responses. However, in this study, gist memory estimation included both vague gist memory retrieval and phantom recollection, without dissociating the two processes. Yet, false memories in young children may not be underpinned by the same processes as false memories in young adults. In the present study, lists of eight words related to a common theme were presented, followed by a retention interval of a few seconds and a recognition task including studied words and related and unrelated distractors. Based on the simplified CR procedure, in the recognition task, participants were asked to identify the type of each probe, which then allowed us to distinguish the contributions of verbatim memory, vague gist memory, phantom recollection, and guessing processes to recognition performance. Each word list to be remembered was composed of four categories of related words. Several categories that were associatively related were presented so that the words repeatedly cued the gist of the list, which should promote the occurrence of false memories. Moreover, the list theme word was given before each list presentation to facilitate the extraction of the gist of each list. Finally, because Abadie and Camos’ (2019) study showed that false memories were more frequent when WM maintenance through articulatory rehearsal was prevented, we manipulated the opportunity to maintain information during the retention interval by asking participants to either perform a concurrent attentionally demanding task that also prevented the use of articulatory rehearsal or a less demanding task without concurrent articulation. The attentional demand of the concurrent task was also manipulated in the present study because information can also be maintained in WM by attentional processes (e.g., Camos et al. 2018); thus, WM maintenance was completely prevented in one condition and not in the other. We expected that false memories of related distractors would occur regardless of the concurrent task and participants’ age, but that they would be more frequent when the concurrent task was highly demanding with concurrent articulation rather than when it was less demanding without concurrent articulation (but see Rousselle et al. 2022, for a similar paradigm with no effect of concurrent task type on false memories), and in young adults than in children. More importantly, we expected that vague gist memory, as previously shown (e.g., Abadie and Camos 2019; Flegal and Reuter-Lorenz 2014; Rousselle et al. 2022), but also phantom recollection (Flegal et al. 2010; Flegal and Reuter-Lorenz 2014), would underlie short-term false memories in adults as well as in young children. Based on the FTT, we also expected verbatim and gist memory to increase with age (e.g., Brainerd and Reyna 2012). Although few studies to date have distinguished the contributions of vague gist memory and phantom recollection to recognition performance in children (Brainerd et al. 2004; Odegard et al. 2008), we assumed that the increase in short-term false memories with age might be underpinned by an increase in both processes. 

## 2. Method

### 2.1. Participants

Thirty-nine children in the 4th and 5th grade recruited from a French elementary school (25 females; *M_age_* = 110.8 months; *SD_age_* = 5.88; range = 100–121) and thirty-five young adults participated in the study (21 females; *M_age_* = 22.7 years; *SD_age_* = 4.7; range = 18–32). All participants were native French speakers. One child and one adult were excluded from the analyses because of their failure to follow instructions. All children and young adults were healthy and predominantly White, mostly from the SUD region in France, in families with middle to higher SES based on the location of the recruitment. This experiment was approved by the local ethics committee. Prior to participation, written informed consent was obtained from the young adults and from the parents for the children. 

### 2.2. Material and Procedure

We developed five eight-word lists. Each included four categories of words (e.g., insect, tree, bird, and fruit category) that were associatively related to a common theme (e.g., forest)^1^. Coane et al. (2016, 2020) showed that categorical plus associative lists elicited higher false memory rates than purely associative lists. Five themes (i.e., forest, market, school, zoo, and sea theme) were selected from those created by Rousselle et al. (2022). Each selected theme was composed of words that were familiar to young children and could be organized into four categories. Half of these categories included the three most prototypical exemplars of the category (e.g., ant, fly, and bee for the “insect” category), and the other half only one exemplar (Cannard et al. 2006). 

**Figure 1:**
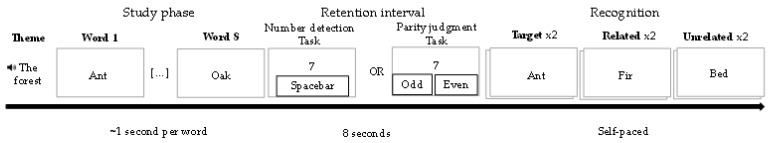
Illustration of the experimental procedure.

Figure 1 illustrates the experimental procedure. In each trial, participants were first presented with the list theme word, followed by each of the eight list words presented sequentially and in random order. All words were presented both auditorily and visually to keep the presentation time of each word similar between children and adults, while maintaining a stimulus on the screen to ensure that participants’ attention remained on the screen. After the presentation of each list, a 500 ms interval accompanied by a sound signal alerted participants to the start of the concurrent task. This task was highly attentionally demanding for half of them, while it was less demanding for the other half. Children and young adults were presented with five and eight digits, respectively. Each digit was presented for 1200 ms for children and 700 ms for young adults with an inter-stimulus interval (ISI) of 300 ms. These parameters were adjusted for age to equalize the cognitive load of this task between the two age groups (e.g., Barrouillet et al. 2009; Gaillard et al. 2011; Gavens and Barrouillet 2004). In the highly demanding condition, all participants had to indicate, for each digit, aloud and by pressing a corresponding key, whether it was even or odd. In the less demanding condition, they only had to press the spacebar each time a digit appeared on the screen (see Rosselet-Jordan et al. 2022 for a similar manipulation). We assumed that WM maintenance through articulatory rehearsal was prevented in the highly demanding condition since participants were required to make a judgment aloud, whereas there was no concurrent articulation in the less demanding condition. Furthermore, we assumed that a parity judgment was more attentionally demanding than a simple detection task (e.g., Barrouillet et al. 2004, 2007). After the concurrent task, all participants performed a recognition task. Three probe types were presented: target probes, which were studied words; related distractors, which were unpresented words semantically related to one of the list categories; and unrelated distractors, which were unpresented words unrelated to the list theme. Two probes of each probe type were presented per trial and participants had to indicate for each of them whether or not it was presented in the study list. When they responded “no”, they were asked whether the probe could be related to one or several studied words. 

At the beginning of the experimental session, participants were first trained on the concurrent task alone and then on the concurrent and memory tasks together. At the end of the experiment, all the children received a medal to thank them for their participation. The experimenter was present throughout the session, but all the instructions were pre-recorded as a video and given by a girl avatar so that they were standardized for all children and the experiment was playful for them.

## 3. Results

### 3.1. Concurrent Task

Concurrent task accuracy was high for both children (81.6%, *SE* = 3.5) and adults (90.5%, *SE* = 1.6), ensuring that participants did not favor the memory task at the expense of poor performance in the concurrent task. 

### 3.2. Memory Accuracy

**Figure 2:**
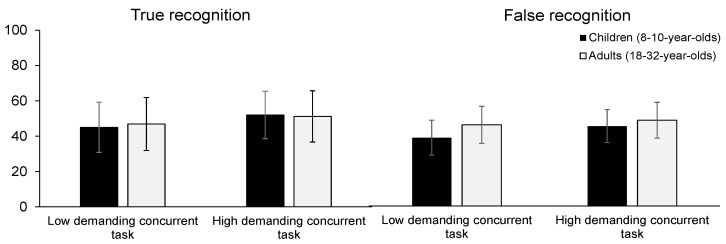
True and false recognition accuracy as a function of age group and attentional demand of the concurrent task. Note: We obtained the discriminability index by subtracting the proportion of false alarms to unrelated probes from the proportion of correct recognition of target probes and the proportion of false alarms to related probes. Error bars represent 95% confidence intervals for mean difference.

We first report the results on true and false recognition. Discriminability indexes were computed on true and false recognition to account for potential developmental differences in “yes-saying” bias (Banks 1970). True and false recognition were conditionalized by subtracting the baseline false alarm rate of unrelated distractors (i.e., responses “yes, this word was in the study list” to unrelated distractors) from the rate of correct recognition of target probes (i.e., responses “yes” to target probes) and from the rate of false recognition of related distractors (i.e., responses “yes” to related distractors). Discriminability indexes for true and false recognition are shown in Figure 2. True and false recognition indexes were significantly higher than chance (*t*(70) = 8.93, *p* < .001, *t*(70) = 9.08, *p* < .001, respectively). We conducted a 2 (age group) × 2 (concurrent task) between-subjects ANOVA on true and false recognition. The analyses were performed with JASP (Version 0.16.3, JASP Team 2022). For true recognition, since Levene’s test for equality of variances was significant (*p* = .02), we used the non-parametric Kruskal–Wallis test, which indicated that neither age group (*H*(1) = 0, *p* = .971) nor concurrent task type (*H*(1) = 3.1, *p* = .078) had a significant impact on true recognition. For false recognition, as predicted, the ANOVA revealed a significant main effect of age group (*F*(1) = 4.54, *p* = .037, ƞ^2^_p_ = .063), the false recognition rate being higher in adults than children. No other effect was significant (*p*_s_ ≥ .071). 

**Figure 3:**
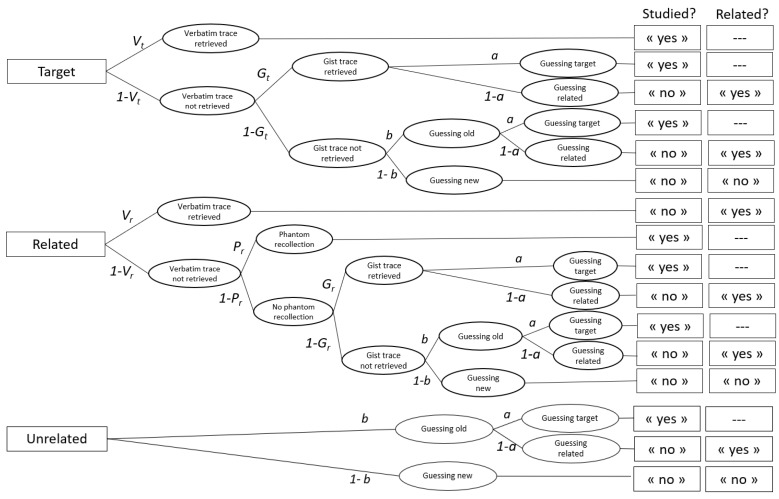
Processing tree for the simplified CR model based on Stahl and Klauer (2009). Note: Rectangles on the left represent probe types, and those on the right represent responses to questions “Was the word in the study list?” and “Was the word related with a word in the study list?”. They are connected by branches that represent the combination of cognitive processes postulated by the model.

Second, the data were analyzed using the simplified CR model (Stahl and Klauer 2009) depicted in Figure 3. The model has five memory parameters, verbatim memory for targets (*V_t_*), gist memory for targets (*G_t_*), verbatim memory for related distractors (*V_r_*), phantom recollection for related distractors (*P_r_*), and gist memory for related distractors (*G_r_*), and two guessing parameters (*a* and *b*). Consider the first tree in Figure 3, which represents the processes occurring when a target probe is presented at test. When its presentation elicits the retrieval of verbatim memory (*V_t_*), it is correctly recognized as a target. When there is no verbatim memory but available gist memory (*G_t_*), participants identify the probe meaning as old, but they cannot remember whether the probe itself or a related word with the same gist was presented in the study phase. They must decide between the responses “yes, this word was on the study list” (i.e., a “target” response) or “no it was not on it, but it is a related word” (i.e., a “related” response). With probability *a*, the probe is recognized as a target, and with probability 1 − *a*, it is recognized as a related distractor. When neither verbatim nor gist memory is available, participants can still guess with the probability *b* that the probe’s meaning is old. A decision between the target or related response is required and captured by the parameter *a* as described above. They can also guess with the probability 1 − *b*, that the probe is neither studied nor related. 

The second tree represents the processes involved when a related distractor is presented at test. When verbatim memory (*V_r_*) is available, it is correctly identified as a related probe. When there is no verbatim memory, a related probe can be experienced as exactly matching a specific presented item and be falsely identified as such (i.e., a phantom recollection) with the probability *P_r_*. Gist memory retrieval (*G_r_*) that lacks such vivid recollective experience leads participants to identify the probe meaning as old but would not allow them to remember whether the probe itself or a related word with the same gist was studied. Guessing processes, as modelled by the parameter *a*, then determined recognition as either a target or a related probe. When no memory traces are available, participants can guess that the probe meaning is old with the probability *b*. Finally, as shown in the third tree, identification of unrelated distractors is based on a combination of guessing processes *a* and *b*.

The simplified CR model has seven parameters, while the data yield only six degrees of freedom (i.e., two free empirical probabilities for targets, related distractors, and unrelated distractors), implying that the model is not identified without additional degrees of freedom or restrictions on (some of) the parameters. The number of free parameters can be reduced by fixing one or more parameters to constant values or by equating two or more parameters. We introduced the restriction *V_r_* = 0 under the assumption that the contribution of the recollection-rejection process (*V_r_*) to the data is negligible. Support for the theoretical and empirical plausibility of this assumption is provided in the validation study of the simplified CR model (Stahl and Klauer 2009). Moreover, memory parameters (*V_t_*, *G_t_*, *P_r_*, *G_r_*) were allowed to vary across the two levels of age group factor, and they were set equally across the concurrent task factor because this manipulation, as we have just seen above, had no effect on our data (see also Rousselle et al. 2022, for similar results). 

As recommended by Schmidt et al. (2022), we conducted a priori power analysis using multiTree (Version 0.47, Moshagen 2010). For the memory parameters of the baseline model under H1, we assume a relatively large difference of .25 between adults and children. The guessing parameters were assumed to resemble the parameter estimates under H0. When using a significance level of α = .05, the power analysis shows that detecting the specified medium effect with a power of 1 − β = .80 requires a total number of observations of *N* = 1179. Hence, the number of observations in our data set (*N* = 2010) was sufficient to detect a difference of .25 in memory parameters between age groups.

Parameter estimations and hypotheses tests were also performed with multiTree. The model including the above restrictions fit the data well (*G*^2^_(d*f* = 8)_ = 8.22, *p* = .41). The goodness-of-fit deteriorated when guessing parameters were set equally across the concurrent task factor (*G*^2^_(d*f* = 12)_ = 21.2, *p* = .047), indicating that this manipulation had an impact on these parameters. Parameter estimates are given in Table 1. Interestingly, as predicted, phantom recollection (parameter *P_r_*) was greater in adults than children (Δ*G*^2^_(d*f* = 1)_ = 10.2, *p* = .001). Phantom recollection was not significantly different from zero in children (Δ*G*^2^_(d*f* = 1)_ = 0.77, *p* = .380), but it was significantly different from zero in young adults (Δ*G*^2^_(d*f* = 1)_ = 16.2, *p* < .001), indicating that reliable phantom recollection can occur even in WM tasks. Moreover, a simpler model without a parameter for phantom recollection did not provide an adequate fit to the data (Δ*G*^2^_(d*f* = 2)_ = 16.9, *p* < .001). Unexpectedly, gist-based false memory (parameter *G_r_*) was not significantly affected by age group (Δ*G*^2^_(d*f* = 1)_ = 1.34, *p* = .246). Verbatim memory for targets (parameter *V_t_*) was greater for young adults than children, as expected (Δ*G*^2^_(d*f* = 1)_ = 5.72, *p* = .017). By contrast, gist memory for targets, parameter *G_t_*, was not significantly affected by age group (Δ*G*^2^_(d*f* = 1)_ = 3.06, *p* = .08). Turning to guessing parameters, parameter *b* was significantly greater for young adults than children (Δ*G*^2^_(d*f* = 2)_ = 9.77, *p* = .008); this was true in the highly demanding condition (Δ*G*^2^_(d*f* = 1)_ = 6.9, *p* = .009) but not in the less demanding condition (Δ*G*^2^_(d*f* = 1)_ = 2.84, *p* = .092). By contrast, parameter *a* was higher in children than in adults (Δ*G*^2^_(d*f* = 2)_ = 6.89, *p* = .003), and this was the case in both the highly and the less demanding condition (Δ*G*^2^_(d*f* = 1)_ = 4.54, *p* = .033, Δ*G*^2^_(d*f* = 1)_ = 6.87, *p* = .009, respectively). 

## 4. Discussion

The present study yielded two outcomes of significance for the study of memory and its development. The first is that phantom recollection can occur in much shorter time frames than previously thought. The second is that children appear to be relatively insensitive to this illusion compared to adults. 

A significant rate of false memories occurred after learning DRM-like lists of eight words, followed by a retention interval of only a few seconds. As in conventional DRM studies, the rate of false recognition of related distractors rivaled the rate of correct recognition of targets. There were no age-related differences in the percentage of correct responses, which was well above chance in both age groups, indicating that the task was well suited to these two groups. We also replicated the developmental reversal classically obtained in the long-term memory literature with a higher proportion of false memories in adults than in children (e.g., Holliday et al. 2011; see Brainerd 2013; Brainerd et al. 2008, for reviews). Using the simplified CR methodology, rather than introspective self-reports as in previous WM studies (e.g., Flegal et al. 2010; Flegal and Reuter-Lorenz 2014), we were able to directly capture the processes underlying recognition responses. Interestingly, in young adults, the results showed that phantom recollection accounted for half of the false memories, whereas only 2% were due to vague gist retrieval (i.e., ((1 − *P_r_*) × *G_r_* × *a*)), and the rest were due to guessing processes. Thus, a significant proportion of young adults’ semantic errors were explained by the conscious retrieval of non-existent details accompanying the related distractor “presentation”. The proportion of responses related to phantom recollection among young adults in the present study was higher than that associated with the *remember* response in Flegal et al.’s (2010; Flegal and Reuter-Lorenz 2014) study, in which the same paradigm but with fewer words (i.e., four) was used. Indeed, in these latter studies, on average, one third of false memories were associated with the *remember* responses, one third with the *know* responses, and one third with the *guess* responses. Moreover, the false memory rate was also doubled in the present study, which can account for the high rate of phantom recollection in young adults. 

The higher rate of false memories obtained in the present experiment compared to previous ones using a similar paradigm with young adults (e.g., Abadie and Camos 2019; Atkins and Reuter-Lorenz 2008; Flegal et al. 2010; Flegal and Reuter-Lorenz 2014) could be explained by the greater number of items to be remembered. In the present experiment, participants were required to maintain eight words. It is possible that they were unable to maintain them in WM because this is beyond its capacity (e.g., Cowan 2001). Many studies have shown that WM capacity increases with age, from an average of four to six letters from age 8 to adulthood (e.g., Dempster 1981, 1985). However, the eight words in each presented list could be grouped into four categories, or chunks, by participants (Chen and Cowan 2009; Cowan et al. 2012). This grouping strategy may have allowed participants to hold them in WM, which may partially explain their good performance on the recognition task. In addition, the large number of words to remember may also have led them to rely more heavily on episodic long-term memory (LTM) processes, which could explain the large number of false memories. Congruently, Abadie and Camos (2019) showed that the rate of false memories increases when maintenance in WM is blocked and thus participants must rely on other processes, such as LTM processes, that lead them to make semantic memory errors. The lack of effect of concurrent task variation in true and false recognition in the present study further supports this hypothesis. This suggests that, contrary to our expectations, information was not maintained in WM in either condition, possibly because the recognition task did not encourage participants to engage in active item maintenance during the retention interval (e.g., Allen et al. 2018; Malmberg 2008; Uittenhove et al. 2019).

Another potential explanation for the large number of false memories obtained in the present experiment could come from the word lists used. Coane et al. (2016, 2020) showed that word lists, equalized in term of backward associative strength with the critical theme word (BAS), that not only shared an associative similarity with the latter but also belonged to the same category (e.g., for the “music” theme, the words “piano” and “guitar”) produced more false memories than purely associative lists (e.g., for the “music” theme, the words “piano” and “radio”), a phenomenon referred to as the feature boost effect. Moreover, Coane et al. (2021) recently reported evidence for a feature boost effect in a short-term memory paradigm. Thus, this effect coming from the addition of extra feature similarity may also occur when the memory test takes place only seconds after encoding. In the present study, the words in each list shared both associative and categorical similarity, whereas purely associative lists have been used in previous studies (e.g., Abadie and Camos 2019; Atkins and Reuter-Lorenz 2008; Flegal et al. 2010; Flegal and Reuter-Lorenz 2014), which may explain the increase in false memories observed here. Such word lists mixing associative and categorical similarity would activate a network of LTM semantic knowledge during their encoding. When related distractors are presented at test, retrieval of verbatim representations of the target words fails, and the sense of familiarity with these distractors is so strong that they are confused with those actually studied, with a strong sense of retrieval of contextual details from their previous presentation when they are probably self-generated words. 

The second important result of our study is that children had fewer false memories than adults. Children’s false memories, however, remained frequent and rivaled their correct recognition rate. Compared to previous studies using the same paradigm in children (Rousselle et al. 2022), children’s false memory rate was much higher in the present study, which can be explained, as for adults, by the increase in the number of words to be remembered, which doubled, and by the use of lists mixing associative and categorical similarity. Interestingly, the processes underlying false memories in children were different from those in adults. Compared to adults, children made significantly fewer false memories based on phantom recollection (16%); they were more driven by vague gist similarity (27%) or guessing (56%). A first reason could be that categorical information depends on abstract and complex knowledge systems, which emerge later in childhood compared to associative or thematic organization. Children would therefore be less prone to the feature boost effect that may underlie the increase in phantom recollection in adults. However, the list themes used were for the most part taken from the material of Rousselle et al. (2022), which was designed for children as young as 4 years of age. In addition, the list theme was given at the outset, and other studies have shown that gist cuing significantly increases false memories in children (Otgaar et al. 2014; Brainerd and Reyna 2012, for a review). Finally, gist-based false memories (i.e., when considering both vague gist memory and phantom recollection) were quite high in children, suggesting that they have some knowledge of semantic similarity between the words. Another potential explanation could be that adults would be more likely to retrieve perceptual details (true or false) associated with the presentation context of an item (e.g., its position in the list, the color of the ink, the shape of the item, etc.) than children aged 8–10 years. This explanation would account not only for the fact that adults made more phantom recollections, but also for the fact that they gave more correct responses based on verbatim retrieval when presented with a target probe (47% in adults vs. 23% in children). Although not on the same age range, a study by Brainerd et al. (2004) also reported a large age increase from early childhood to adolescence in the tendency of target probes and semantically related distractors to provoke realistic mental reinstatement of their presentation or of the presentation of their corresponding target. Together, Brainerd et al.’s results and ours suggest a significant developmental shift from childhood to adulthood in memory representations that are initially vague and become more vivid with more real or illusory contextual details during development. 

The present study raises two important questions that need to be addressed in future research. The first concerns the impact of the mechanisms of information maintenance in WM, whose efficiency increases from childhood to adulthood (Barrouillet et al. 2009; Camos and Barrouillet 2011; Gaillard et al. 2011), on these vivid short-term false memories. The second is the developmental trajectory of these memory distortions, which remains to be rigorously investigated within a single study covering different ages from early childhood to adulthood.

## 5. Conclusions

To conclude, the present study showed, for the first time, that a high level of phantom recollection can occur in a WM task with lists of a few items and a study-test interval of a few seconds. Importantly, these vivid short-term false memories increase dramatically with age from 8–10 years to adulthood. These findings again add to the extensive evidence against the intuitive assumption that children’s testimony is inherently more infected with false memories than adults’.

## Figures and Tables

**Table 1:** Estimates (and 95% confidence intervals) of simplified CR model parameters.

	Children (8–10-Year-Olds)		Adults (18–32-Year-Olds)	
	Less Demanding Concurrent Task	Highly Demanding Concurrent Task	Less Demanding Concurrent Task	Highly Demanding Concurrent Task
*V_t_*	.23 [−.07, .52]		.47 [.35, .58]	
*G_t_*	.93 [.89, .97]		.99 [.98, .10]	
*P_r_*	.16 [−.13, .46]		.46 [.35, .56]	
*G*_r_	.86 [.80, .92]		.93 [.89, .97]	
*b*	.08 [.04, .12]	.01 [−.01, .0.3]	.04 [.01, .06]	.07 [.03, .10]
*a*	.38 [.14, .61]	.39 [.13, .64]	0 [−.22, .22]	.08 [−.07, .23]

Note: *V_t_* = verbatim memory for targets; *G_t_* = gist memory for targets; *P_r_* = phantom recollection; *G_r_* = gist memory for related distractors; *b* = probability of guessing that an item is either a target or a related probe; *a* = probability of guessing ‘target’.

## Data Availability

Data are available on the OSF at https://osf.io/wc5g8/?view_only=4747867314604243986cc5b13a826cdb (accessed on 27 March 2023).

## Note

1 The word lists used are available on the OSF at https://osf.io/wc5g8/?view_only=4747867314604243986cc5b13a826cdb (accessed on 27 March 2023).

## References

[B1-jintelligence-11-00067] Abadie Marlène, Camos Valérie (2019). False memory at short and long term. Journal of Experimental Psychology: General.

[B2-jintelligence-11-00067] Allen Richard J., Hitch Graham J., Baddeley Alan D. (2018). Exploring the sentence advantage in working memory: Insights from serial recall and recognition. Quarterly Journal of Experimental Psychology.

[B3-jintelligence-11-00067] Atkins Alexandra S., Reuter-Lorenz Patricia A. (2008). False working memories? Semantic distortion in a mere 4 seconds. Memory and cognition.

[B4-jintelligence-11-00067] Banks William P. (1970). Signal detection theory and human memory. Psychological Bulletin.

[B5-jintelligence-11-00067] Barrouillet Pierre, Gavens Nathalie, Vergauwe Evie, Gaillard Vinciane, Camos Valérie (2009). Working memory span development: A time-based resource-sharing model account. Developmental Psychology.

[B6-jintelligence-11-00067] Barrouillet Pierre, Bernardin Sophie, Camos Valérie (2004). Time constraints and resource sharing in adults’ working memory spans. Journal of Experimental Psychology: General.

[B7-jintelligence-11-00067] Barrouillet Pierre, Bernardin Sophie, Portrat Sophie, Vergauwe Evie, Camos Valérie (2007). Time and cognitive load in working memory. Journal of Experimental Psychology: Learning, Memory, and Cognition.

[B8-jintelligence-11-00067] Brainerd Charles J. (2013). Developmental Reversals in False Memory: A New Look at the Reliability of Children’s Evidence. Current Directions in Psychological Science.

[B9-jintelligence-11-00067] Brainerd Charles J., Reyna Valerie F. (1998). When things that never happened are easier to remember than things that did. Psychological Science.

[B10-jintelligence-11-00067] Brainerd Charles J., Reyna Valerie F. (2012). Reliability of children’s testimony in the era of developmental reversals. Developmental Review.

[B11-jintelligence-11-00067] Brainerd Charles J., Reyna Valerie F. (2015). Fuzzy-trace theory and lifespan cognitive development. Developmental Review.

[B12-jintelligence-11-00067] Brainerd Charles J., Reyna Valerie F. (2018). Complementarity in false memory illusions. Journal of Experimental Psychology: General.

[B13-jintelligence-11-00067] Brainerd Charles J., Payne David G., Reyna Valerie F. (2003). Phantom Recall. Journal of Memory and Language.

[B14-jintelligence-11-00067] Brainerd Charles J., Holliday Robyn E., Reyna Valerie F. (2004). Behavioral measurement of remembering phenomenologies: So simple a child can do it. Child Development.

[B15-jintelligence-11-00067] Brainerd Charles J., Wright Ron, Reyna Valerie F., Mojardin Ambrocio H. (2001). Conjoint recognition and phantom recollection. Journal of Experimental Psychology: Learning, Memory, and Cognition.

[B16-jintelligence-11-00067] Brainerd Charles J., Reyna Valerie F., Mojardin Ambrocio H. (1999). Conjoint recognition. Psychological review.

[B17-jintelligence-11-00067] Brainerd Charles J., Reyna Valerie F., Zember Eric (2011). Theoretical and forensic implications of developmental studies of the DRM illusion. Memory & Cognition.

[B18-jintelligence-11-00067] Brainerd Charles J., Reyna Valerie F., Holliday Robyn E. (2018). Developmental reversals in false memory: Development is complementary, not compensatory. Developmental Psychology.

[B19-jintelligence-11-00067] Brainerd Charles J., Reyna Valerie F., Ceci Stephen J. (2008). Developmental reversals in false memory: A review of data and theory. Psychological Bulletin.

[B20-jintelligence-11-00067] Camos Valérie, Barrouillet Pierre (2011). Developmental change in working memory strategies: From passive maintenance to active refreshing. Developmental Psychology.

[B21-jintelligence-11-00067] Camos Valérie, Johnson Matthew, Loaiza Vanessa, Portrat Sophie, Souza Alessandra, Vergauwe Evie (2018). What is attentional refreshing in working memory?. Annals of the New York Academy of Sciences.

[B22-jintelligence-11-00067] Camos Valérie (2015). Storing verbal information in working memory. Current Directions in Psychological Science.

[B23-jintelligence-11-00067] Camos Valérie (2017). Domain-specific vs. domain-general maintenance in working memory: Reconciliation within the time-based resource sharing model. Psychology of Learning and Motivation.

[B24-jintelligence-11-00067] Cannard Christine, Bonthoux Françoise, Blaye Agnès, Scheuner Nelly, Schreiber Anne-Caroline, Trinquart Jacques (2006). BD2I: Normes sur l’identification de 274 images d’objets et leur mise en relation chez l’enfant français de 3 à 8 ans. Année Psychologique.

[B25-jintelligence-11-00067] Chang Minyu, Brainerd Charles J. (2021). Semantic and phonological false memory: A review of theory and data. Journal of Memory and Language.

[B26-jintelligence-11-00067] Chen Zhijian, Cowan Nelson (2009). Core verbal working-memory capacity: The limit in words retained without covert articulation. The Quarterly Journal of Experimental Psychology.

[B27-jintelligence-11-00067] Coane Jennifer H., McBride Dawn M., Xu Shuofeng (2020). The feature boost in false memory: The roles of monitoring and critical item identifiability. Memory.

[B28-jintelligence-11-00067] Coane Jennifer H., McBride Dawn M., Huff Mark J., Chang Kai, Marsh Elizabeth M., Smith Kendal A. (2021). Manipulation of list type in the DRM paradigm: A review of how structural and conceptual similarity affect false memory. Frontiers in Psychology.

[B29-jintelligence-11-00067] Coane Jennifer H., McBride Dawn M., Termonen Miia-Liisa, Cutting J. Cooper (2016). Categorical and associative relations increase false memory relative to purely associative relations. Memory & Cognition.

[B30-jintelligence-11-00067] Cowan Nelson (2001). The magical number 4 in short-term memory: A reconsideration of mental storage capacity. Behavioral and Brain Sciences.

[B31-jintelligence-11-00067] Cowan Nelson, Rouder Jeffrey N., Blume Christopher L., Saults J. Scott (2012). Models of verbal working memory capacity: What does it take to make them work?. Psychological Review.

[B32-jintelligence-11-00067] Deese James (1959). On the prediction of occurrence of particular verbal intrusions in immediate recall. Journal of Experimental Psychology.

[B33-jintelligence-11-00067] Dempster Frank N. (1981). Memory span: Sources of individual and developmental differences. Psychological Bulletin.

[B34-jintelligence-11-00067] Dempster Frank N., Brainerd Charles J., Pressley Michael (1985). Short-term memory development in childhood and adolescence. Basic processes in Memory Development: Progress in Cognitive Development Research.

[B35-jintelligence-11-00067] Flegal Kristin E., Atkins Alexandra S., Reuter-Lorenz Patricia A. (2010). False memories seconds later: The rapid and compelling onset of illusory recognition. Journal of Experimental Psychology: Learning, Memory, and Cognition.

[B36-jintelligence-11-00067] Flegal Kristin E., Reuter-Lorenz Patricia A. (2014). Get the gist? The effects of processing depth on false recognition in short-term and long-term memory. Memory and Cognition.

[B37-jintelligence-11-00067] Gaillard Vinciane, Barrouillet Pierre, Jarrold Christopher, Camos Valérie (2011). Developmental differences in working memory: Where do they come from?. Journal of Experimental Child Psychology.

[B38-jintelligence-11-00067] Gallo David A., McDermott Kathleen B., Pereer Jenny M., Roediger Henry L. (2001). Modality effects in false recall and false recognition. Journal of Experimental Psychology: Learning, Memory, and Cognition.

[B39-jintelligence-11-00067] Gavens Nathalie, Barrouillet Pierre (2004). Delays of retention, processing efficiency, and attentional resources in working memory span development. Journal of Memory and Language.

[B40-jintelligence-11-00067] Geraci Lisa, McCabe David P. (2006). Examining the basis for illusory recollection: The role of remember/know instructions. Psychonomic Bulletin & Review.

[B41-jintelligence-11-00067] Holliday Robyn E., Brainerd Charles J., Reyna Valerie F. (2011). Developmental reversals in false memory: Now you see them, now you don’t!. Developmental Psychology.

[B42-jintelligence-11-00067] Israel Lana, Schacter Daniel L. (1997). Pictorial encoding reduces false recognition of semantic associates. Psychonomic Bulletin & Review.

[B43-jintelligence-11-00067] JASP Team (2022). JASP.

[B44-jintelligence-11-00067] Kim Hongkeun, Cabeza Roberto (2007). Trusting our memories: Dissociating the neural correlates of confidence in veridical and illusory memories. Journal of Neuroscience.

[B45-jintelligence-11-00067] Malmberg Kenneth J. (2008). Recognition memory: A review of the critical findings and an integrated theory for relating them. Cognitive Psychology.

[B46-jintelligence-11-00067] Marche Tammy A., Brainerd Charles J. (2012). The role of phantom recollection in false recall. Memory & Cognition.

[B47-jintelligence-11-00067] Moshagen Morten (2010). MultiTree: A computer program for the analysis of multinomial processing tree models. Behavior Research Methods.

[B48-jintelligence-11-00067] Odegard Timothy N., Holliday Robyn E., Brainerd Charles J., Reyna Valerie F. (2008). Attention to global gist processing eliminates age effects in false memories. Journal of Experimental Child Psychology.

[B49-jintelligence-11-00067] Otgaar Henry, Howe Mark L., Peters Maarten, Smeets Tom, Moritz Steffen (2014). The production of spontaneous false memories across childhood. Journal of Experimental Child Psychology.

[B50-jintelligence-11-00067] Payne David G., Elie Claude J., Blackwell Jason. M., Neuschatz Jeffrey S. (1996). Memory illusions: Recalling, recognizing, and recollecting events that never occurred. Journal of Memory and Language.

[B51-jintelligence-11-00067] Pesta Bryan J., Murphy Martin D., Sanders Raymond E. (2001). Are emotionally charged lures immune to false memory?. Journal of Experimental Psychology: Learning, Memory, and Cognition.

[B52-jintelligence-11-00067] Roediger Henry L., McDermott Kathleen B. (1995). Creating False Memories: Remembering Words Not Presented in Lists. Journal of Experimental Psychology: Learning, Memory, and Cognition.

[B53-jintelligence-11-00067] Rosselet-Jordan Fiona Laura, Abadie Marlène, Marz-Elsig Stéphanie, Camos Valérie (2022). Role of attention in the associative relatedness effect in verbal working memory: Behavioral and chronometric perspectives. Journal of Experimental Psychology: Learning, Memory, and Cognition.

[B54-jintelligence-11-00067] Rousselle Manon, Abadie Marlène, Blaye Agnès, Camos Valérie (2022). Children’s gist-based false memory in working memory tasks. Developmental Psychology.

[B55-jintelligence-11-00067] Schmidt Oliver, Erdfelder Edgar, Heck Daniel W. (2022). Tutorial on Multinomial Processing Tree Modeling: How to Develop, Test, and Extend MPT Models. PsyArXiv.

[B56-jintelligence-11-00067] Stahl Christoph, Klauer Karl Christoph (2009). Measuring phantom recollection in the simplified conjoint recognition paradigm. Journal of Memory and Language.

[B57-jintelligence-11-00067] Uittenhove Kim, Chaabi Lina, Camos Valérie, Barrouillet Pierre (2019). Is working memory storage intrinsically domain-specific?. Journal of Experimental Psychology: General.

